# Discriminative variable subsets in Bayesian classification with mixture models, with application in flow cytometry studies

**DOI:** 10.1093/biostatistics/kxv021

**Published:** 2015-06-03

**Authors:** Lin Lin, Cliburn Chan, Mike West

**Affiliations:** Vaccine and Infectious Disease Division, Fred Hutchinson Cancer Research Center, Seattle, WA 98109, USA; Biostatistics & Bioinformatics, Duke University Medical Center, Durham, NC 27710-2721, and Department of Statistical Science, Duke University, Durham, NC 27708-0251, USA; Department of Statistical Science, Duke University, Durham, NC 27708-0251, USA

**Keywords:** Bayesian expectation–maximization, Bayesian mixture models, Classification error rates, Concordance of densities, Dirichlet process mixtures, Discriminative information measure, Discriminative threshold probabilities, Flow cytometry data, Non-Gaussian component mixtures, Variable subset selection

## Abstract

We discuss the evaluation of subsets of variables for the discriminative evidence they provide in multivariate mixture modeling for classification. The novel development of Bayesian classification analysis presented is partly motivated by problems of design and selection of variables in biomolecular studies, particularly involving widely used assays of large-scale single-cell data generated using flow cytometry technology. For such studies and for mixture modeling generally, we define discriminative analysis that overlays fitted mixture models using a natural measure of concordance between mixture component densities, and define an effective and computationally feasible method for assessing and prioritizing subsets of variables according to their roles in discrimination of one or more mixture components. We relate the new discriminative information measures to Bayesian classification probabilities and error rates, and exemplify their use in Bayesian analysis of Dirichlet process mixture models fitted via Markov chain Monte Carlo methods as well as using a novel Bayesian expectation–maximization algorithm. We present a series of theoretical and simulated data examples to fix concepts and exhibit the utility of the approach, and compare with prior approaches. We demonstrate application in the context of automatic classification and discriminative variable selection in high-throughput systems biology using large flow cytometry datasets.

## 1. Introduction

We are interested in the general question of identifying subsets of variables that play roles in discrimination of subpopulations in the context of multivariate mixture modeling. We are particularly concerned with applications of mixture models to increasingly large samples such as arise in single-cell biomolecular studies typified by current, very widely used flow cytometry technology. In such studies, the samples sizes are large (hundreds of thousands to several millions) and the numbers of mixture components representing meaningful subpopulations can run into the several hundreds. Typified by studies of vaccine design and immune response in multiple areas involving clinical studies of infectious diseases, there is a need for formal statistical methods to guide identification and prioritization of molecular markers both as end-points to variable selection studies and for follow-on confirmatory experiments. The challenge to statistical methodology is that of creating valid, effective, and computationally accessible approaches to variable subset assessment and prioritization with regard to the ability of each subset to discriminate one or more subpopulations from the rest.

Routinely applied biological cell assays using flow cytometry generate multiple data sets with sample sizes (numbers of cells) in the hundreds of thousands or millions and involving $10$–$20$ variables (cell surface markers) allowing interrogation of cell population heterogeneity. Mixture modeling approaches (e.g., Boedigheimer and Ferbas, 2008; Lo *and others*, 2008; Chan *and others*, 2008; Finak *and others*, 2009; Pyne *and others*, 2009) are now central in studies to automatically identify different cell populations, a necessary step before applying flow cytometric data to design of new studies, e.g., correlation with possible clinical outcomes of interest. Automated mixture model fitting overlaid with an effective approach to discriminative analysis to prioritize subsets of variables can be expected to have a major impact on advancing statistical work in this area, among others.

This is our setting and challenge: Given a mixture model previously fitted (in our cases, using Bayesian methods via Bayesian EM and/or Markov chain Monte Carlo methods), formalize and implement an effective Bayesian/decision analytic approach to prioritizing variables in their ability to discriminate each practically relevant mixture component from the rest.

Notationally, we consider a $p-$dimensional, $C-$component mixture distribution with density function

(1.1) \[g(x) \equiv g(x|\Theta ) = \sum _{c=1}^C \alpha _c f_c(x|\theta _c),\]

where each subpopulation density $f_c$ has its own parameters $\theta _c$ and component probability $\alpha _c, (c=1,\ldots ,C),$ and $\Theta = \{C,\alpha _{1:C},\theta _{1:C}\}$ is the full set of parameters. Based on fitting the model to an observed data set, we address the following questions. For each component $c:$

1. Which subsets of the $p$ variables, if any, contribute in meaningful ways to discrimination of $f_c$ from the other components?
2. Are there variables that are irrelevant to discrimination of $f_c$?
3. Are there single or small subsets of variables that characterize $f_c$ alone and play no roles in discriminating other components?
4. Can we rank subsets of variables by their discriminative ability with respect to $f_c?$

The general questions relate to variable selection in mixtures. We address the discriminative analysis following model fitting: we assume that the model is available with either plug-in parameter estimates or Markov chain Monte Carlo based posterior samples, e.g., Lavine and West (1992), West (1992, 1997), Dellaportas and Papageorgiou (2006), Frühwirth-Schnatter and Pyne (2010) and aim to then interrogate the model to evaluate the discriminative roles of different subsets of variables. As part of this, we directly address the issue that very different subsets of variables may play roles in differentiating different mixture components. Also, we are keen to define computationally effective approaches so as to enable access and routine use, and an ability to scale to larger models.

We provide theoretical development and then examples in Bayesian mixture models using standard truncated Dirichlet process mixtures. The analysis is quite general, but this Bayesian normal mixture framework, e.g., Escobar and West (1995), MacEachern and Müller (1998), Ishwaran and James (2001), and Müller and Quintana (2004) will be familiar to many readers; among other things, it offers an approach to handling uncertainty about $C$ subject to a specified large upper bound.

Our examples and much applied interest lies in mixture models where each component density $f_c$ may have a non-Gaussian form. One effective approach is to fit an encompassing mixture of Gaussians and then aggregate subsets of the fitted densities; that is, each $f_c$ is itself represented as a mixture of, typically, a small number of Gaussians, e.g., Chan *and others* (2008) and Finak *and others* (2009). Supplementary material available at *Biostatistics* online summarizes Bayesian computational methods for Gaussian mixtures and technical details of the subsequent construction of non-Gaussian subpopulation densities, as well as other technical details. Our computational work also introduces a new Bayesian expectation–maximization algorithm for truncated Dirichlet process mixtures, while the MCMC analysis exploits the most effective component relabeling approach Cron and West (2011). Both optimization and simulation analyses utilize efficient parallel implementations of Bayesian computations for these mixture models Suchard *and others* (2010).

## 2. Discriminative information

### 2.1. Classification

In the mixture model of equation (1.1), focus on one of the component distributions $c$. For notational clarity here write $f_c(x)=f_c(x|\theta _c),$ the dependence on parameters being implicit. The mixture pdf is then

(2.1) \[g(x) = \sum _{c=1}^C \alpha _c f_c(x) = \alpha _c f_c(x) + (1-\alpha _c) f_{-c}(x),\]

where $f_{-c}(x)$ is the conditional mixture

(2.2) \[f_{-c}(x)= \frac 1{(1-\alpha _c)}\sum _{a=1:C, a\ne c} \alpha _a f_a(x).\]

We will also interpret this notation as extending to $c$ being a *set* of components, for contexts when we want to compare discrimination of a set/collection of clusters—or subpopulations-from the others; the notation obviously encompasses this.

Now suppose we record an observation at the point $x$ in the sample space with no additional information about its genesis. The classification probability for component $c$—the probability that this case in fact arose from that component (or set of components) — is then simply the posterior probability $\alpha _c^* (x) = \alpha _c f_c(x)/g(x).$ Any hard classification rule chooses to classify $x$ as coming from component (group or cluster) $c$ if $\alpha _c^* (x)$ is large enough, i.e., if $\alpha _c^* (x)>\tau$ for some chosen threshold $\tau .$ Now note that $\alpha _c^* (x)>\tau$ if any only if, $V_{c,\tau }(x)>0$ where

(2.3) \[V_{c,\tau }(x) = \alpha _c f_c(x) - \tau g(x) = \alpha _c(1-\tau ) f_c(x) - (1-\alpha _c)\tau f_{-c}(x).\]

**Definition 1**

As a function of $x$ for given component $c$ and classification probability threshold $\tau , V_{c,\tau }(x)$ in (2.3) is the *classifier* for component $c,$ determining classification boundaries/regions in the sample space.

### 2.2. Discriminative information measures of evidence

*Cases when $x\sim f_c:$* Assume we know that a specific observation $x$ actually arises from component $c,$ i.e., $x\sim f_c$. In such a case, larger values of $V_{c,\tau }(x)$ are desirable to generate high rates of true-positive classifications. We see that

(2.4) \[E(V_{c,\tau }(x) | x\sim f_c) = \alpha _c(1-\tau )\delta _{c,c} - (1-\alpha _c)\tau \delta _{c,-c,}\]

where

(2.5) \[\delta _{a,b} = \int f_a(x)f_b(x)\,{\rm d}x\]

for any two distributions with pdfs $f_a, f_b.$ Note that $\delta _{a,b} = E_b[f_a(x)]$ where the expectation is over $x\sim f_b(\cdot ),$ and the measure is symmetric in $a,b$. The number $\delta _{a,b}$ is a natural measure of agreement, overlap or *concordance* between the two distributions. This measure of concordance takes higher values when $f_a,\ f_b$ are closely similar, is maximized when the densities agree exactly, and otherwise decays towards zero as the densities become more separated. Concordance was discussed as the basis of a similarity distance between densities by Scott and Szewczyk (2001), for example. In the mixture context, assessing how different component $f_c$ is to the set of remaining components of the mixture, it is therefore intuitively natural that the concordance $\delta _{c,-c}$ arises as in (2.4).

Continuing under the true-positive assumption that $x\sim f_c,$ we see that $E(V_{c,\tau }(x) | x\sim f_c)>0$ implies and is implied from (2.4) by

(2.6) \[\Delta _c \lt \frac {\alpha _c}{(1-\alpha _c)}\frac {(1-\tau )}{\tau },\]

where

(2.7) \[\Delta _c = \frac {\delta _{c,-c}}{\delta _{c,c}} = \frac {\int f_c(x)f_{-c}(x)\,{\rm d}x}{\int f_c(x)^2\,{\rm d}x}.\]

*Cases when $x\sim f_{-c}$*: In the complementary case when $x$ actually generated from $f_{-c},$ then for any specified classification probability threshold $\tau ,$ small values of $V_{c,\tau }(x)$ below zero are desirable in order to appropriately classify $x$ with a high success rate. In other words, $V_{c,\tau }(x)$ should be large in terms of its absolute value. A similar argument to that above then yields $E(V_{c,\tau }(x) | x\sim f_{-c})\lt 0$ if, and only if,

(2.8) \[\Delta _{-c} \lt \frac {(1-\alpha _c)}{\alpha _c}\frac {\tau }{(1-\tau )},\]

where $\Delta _{-c}$ is the analogous discriminative information measure of evidence (DIME) for components $-c;$ that is,

(2.9) \[\Delta _{-c} = \frac {\delta _{c,-c}}{\delta _{-c,-c}} =\frac {\int f_c(x)f_{-c}(x)\,{\rm d}x}{\int f_{-c}(x)^2\,{\rm d}x}.\]

**Definition 2**

For any component $c,$ the number $\Delta _c$ defined in (2.7) is the true-positive *discriminative information measure of evidence $($DIME$)$* for component $c.$ The number $\Delta _{-c}$ of (2.9) is the corresponding true-negative DIME value for component $c.$ In comparing discrimination based on different subsets of variables, we will modify the notation to make explicit which variables are used. For any subset of variables $h \subseteq \{1:p\},$ when restricting to the mixture distribution on only the $h$ margin, we denote the DIME values by $\Delta _c(h),\ \Delta _{-c}(h).$

The two DIME values for any component $c$ are standardized, directional versions of the basic concordance measure $\delta _{c,-c}.$ Small values imply good discrimination. Note also that they are measures on a likelihood ratio scale, and so are easily interpretable measures of pure discrimination. Specifically for positive discrimination, (2.6) shows that the DIME value $\Delta _c$ is in fact a likelihood ratio, i.e., a Bayes’ factor, that maps prior odds $\alpha _c/(1-\alpha _c)$ on component $c$ to an implied posterior odds ratio of at least $\tau /(1-\tau )$ based on a Bayes’ factor of $1/\Delta _c.$ Similar comments apply to the role and interpretation of $\Delta _{-c}.$

### 2.3. DIME and classification performance

Given a mixture, we can directly and simply compute the DIME values $\Delta _c,\Delta _{-c}$ as numerical summaries of discrimination of component $c$ from the rest, with their interpretations as Bayes’ factors (likelihood ratios). They nicely quantify discrimination between mixture components decoupled completely from prior probabilities of mixture components and hence from classification rates. Further development now relates them to classification performance in the context of the overall mixture model now involving the prior probabilities $\alpha _c.$

Note that classifying any future $x$ value as coming from component $c$ based on $\alpha _c^* (x)>\tau$ induces a classifier $V_{c,\tau }(x)$ whose expectation under $f_c$ is positive when, from equations (2.6) and (2.7),

(2.10) \[\tau \lt \tau _{c+ }\quad \textrm {where}\ \tau _{c+ } = \frac {\alpha _c}{\alpha _c + (1-\alpha _c) \Delta _c}.\]

Similarly, the expected classifier is negative under $f_{-c}$ when, from equations (2.8) and (2.9),

(2.11) \[\tau >\tau _{c-}\quad \textrm {where}\ \tau _{c-} = \frac {\alpha _c\Delta _{-c}}{1-\alpha _c+ \alpha _c\Delta _{-c}}.\]

The key equations (2.10) and (2.11) map the DIME measures and prior probabilities to easily computable classification thresholds on the interpretable probability scale. It turns out that we can further interpret these thresholds in connection with classification performance measured by the theoretical expected posterior classification probabilities, namely

\[\alpha _{c+ } = E(\alpha _c^* (x) | x\sim f_c)\quad \textrm {and}\quad \alpha _{c-} = E(\alpha _c^* (x) | x\sim f_{-c})\]

where, for all $c=1:C, \alpha _c^* (x) = \alpha _c f_c(x) / g(x).$ Refer to $\alpha _{c+ }$ as the expected true-positive classification rate, and $\alpha _{c-}$ as the expected false-positive rate^1^. In simple examples, these rates can be approximately computed by simulation, e.g., by simple importance sampling. However, computing their values exactly is impossible and estimating them a standing issue in classification with mixtures.

It turns out that the trivially computed classification probability bounds $\tau _{c+ }, \tau _{c-}$ are simple, direct first-order approximations to $\alpha _{c+ }, \alpha _{c-}$, respectively, as follows. For the former, simply note that $\alpha _{c+ } = E[\alpha _c f_c(x) / g(x) | x\sim f_c]$, the expectation of the ratio of $\alpha _c f_c(x)$ to $g(x).$ Using the first-order approximation given by the ratio of the two corresponding expectations yields

\[\alpha _{c+ } \approx \frac {E[\alpha _c f_c(x) | x\sim f_c]}{E[g(x) | x\sim f_c]} = \frac { \alpha _c \delta _{c,c} }{ \alpha _c \delta _{c,c} + (1-\alpha _c) \delta _{c,-c}} = \tau _{c+ }.\]

We similarly deduce $\alpha _{c-} \approx \tau _{c-}$, with details left to the reader. Section 5 of Supplementary material available at *Biostatistics* online shows high accuracy of the approximation works across practically relevant contexts.

**Definition 3**

The quantities $\tau _{c+ }$ and $\tau _{c-}$ are referred to as the true- and false-positive *discriminative threshold probabilities* for classification into component $c$ of the mixture. As with DIME values in Definition 2, when comparing discrimination based on different subsets of variables, we will make explicit in the notation which variables are used. For any subset of variables $h \subseteq \{1:p\},$ when restricting to the mixture distribution on only the $h$ margin, we denote the discriminative threshold probabilities by $\tau _{c+ }(h),\ \tau _{c-}(h).$

This represents an advance in practical evaluation of classification performance using mixtures; direct evaluation of expected classification rates is simply infeasible, while these trivially computed discriminative threshold probabilities are easy to compute based on DIME measures and prior probabilities. In addition to the bounds they provide for classification, they are seen to also define estimates of expected classification rates.

## 3. Practical discrimination

### 3.1. Overview

The above theoretical and conceptual developments generate advances in terms of efficient computation of bounds related to classification rates. Our main interest here is in the practical use of this to evaluate the discriminative information provided by different subsets of variable in connection with one or any of the mixture components. We can now do this in both relative and absolute senses; DIME measures are likelihood ratios that provide relative assessments across variable subsets, while the derived discriminative threshold probabilities provide absolute comparisons on the probability scale. Practically, small values of $\Delta _c, \Delta _{-c}$ are desirable as they will lead to higher sensitivity and specificity in classification of cases as coming from component $c.$ Correspondingly, high values of $\tau _{c+ }$ and low values of $\tau _{c-}$ indicate good discrimination of component $c$ from the rest concerning true-positive and false-positive rates, respectively. These easily computed bounds that depend naturally on concordance between densities can therefore compare and absolutely quantify discriminative abilities of differing subsets of variables within the same overall joint mixture model.

Given a specified mixture on the full set of $p$-variables in $x,$ we will just directly marginalize to the variable subset $h$ to compute DIME values and discrimination probabilities for each component $c=1:C.$ This way the issue of evaluating discriminative subsets can be carried out for all components and any selected subsets of variables based on the fitted model, without refitting. By exploring subsets $h,$ we can automatically generate ranked sets of variables for each component and address the questions above. Notice that, at an extreme, if one or a subset of variables $h$ is independent of the rest and has the same distribution over all components, then $\Delta _c(h)$ will take the same values when computed in the mixture model analysis with or without those variables, showing their irrelevance.

### 3.2. Evaluations on variable subsets

For each component $c=1:C$ and a given subset of variables $h,$ consider the quantity

(3.1) \[A_c(h) = \alpha _c \tau _{c+ }(h) + (1-\alpha _c) (1-\tau _{c-}(h)).\]

This is the natural prior/base-rate weighted average of discriminative threshold probabilities for classification into component $c,$ an overall classification rate summary. Note that it is on the absolute probability scale so differences across different subsets $h$ can be easily interpreted.

**Definition 4**

For each component $c=1:C,$ the quantity $A_c(h)$ in (3.1) is referred to as the *aggregate discriminative accuracy measure* for component $c$ in the marginal mixture distribution for variable subset $h\subseteq \{1:p\}.$

Given the mixture of (1.1) on the full set of $p$ variables, we can directly extract the implied marginal mixture on any subset of variables $h\subseteq \{1:p \}$ for discriminative evaluation of components based on only that subset ($A_c(h)$). When $p$ is small, we can directly evaluate all possible $2^p$ variable subsets. For moderate and larger $p,$ this is of course infeasible and some form of guided search over variable subsets is needed. Forward (Section 7 of Supplementary material available at *Biostatistics* online) and/or backward selection methods are obvious first steps. Our examples and application below utilize a first, direct forward search method as follows. In higher-dimensional problems, it would be natural to utilize ideas of stochastic search methods as used for exploring variable subsets in regression and for graphical model search (e.g., Jones *and others*, 2005; Hans *and others*, 2007), but this is beyond our scope here.

### 3.3. Model fitting and estimation of discriminative measures

All of the above theoretical development has assumed a given mixture model—that is, a given set of values of $C$ and full knowledge of the $\alpha _c$ and all model densities $f_c(x)=f_c(x|\Theta _c)$ in (1.1). In practice, we perform inference on all model parameters and this translates to estimation of the DIME, discriminative threshold probabilities and discriminative accuracy measures. Our Bayesian analysis in examples here uses standard truncated Dirichlet process mixtures of multivariate normal distributions. This context involves fixing a (large) upper bound on the number of normal components in the mixture, and then developing the posterior over all model parameters, including the effective number of components, based on any given data set. Summary details and references are given in Supplementary material available at *Biostatistics* online.

## 4. Synthetic data example

Two proof-of-principle examples to demonstrate the DIME-based analysis are in Section 8 of Supplementary material available at *Biostatistics* online. Here, we show one example that strongly illustrates the ability of DIME-based analysis to dissect component-specific discriminatory variables from the rest, comparing the analysis with the ridgeline-based separability measure (RSM) of Lee and Li (2012), which appears to be the most directly related approach to discriminative assessment. RSM, which measures the pairwise separability between the modes of any two clusters, has been shown to be superior to earlier methods including the scatter separability criterion (SSC) (Dy and Brodley, 2004). RSM is a global measure that selects one subset of variables for all components, and it has an explicit analytic expression only in cases of two component normal mixtures with identity covariance matrices. Logistically, we follow the forward selected strategy and recommendations in Lee and Li (2012), evaluating variables to add to a current discriminatory subset if the increase in RSM exceeds 0.01 at each step, and stopping otherwise. MCMC analyses were initialized at the Bayesian EM-based posterior modes, and we generated posterior simulations of size 10,000 following additional burn-in iterates. For most direct comparison, RSM measures were evaluated using mixture model parameters estimated by MCMC-based posterior means.

We generated a synthetic sample of 6000 from an $8$-dimensional mixture of normals. The mixture has four components of proportions $(0.3, 0.3, 0.3, 0.1)$ and with mean vectors $(7,0,0,0,0,0,0,5), (5,5,5,0,0,0,0,0), (0,0,0,5,5,5,0,0),$ and $(0,0,0,0,0,0,0,0).$ The covariance matrix of component 1 is $\textrm {diag}(1,5,5,5,5,5,5,5);$ for components 2 and 3, the covariance matrix entries for the subsets of variables having non-zero means are

**Fig. 1.**
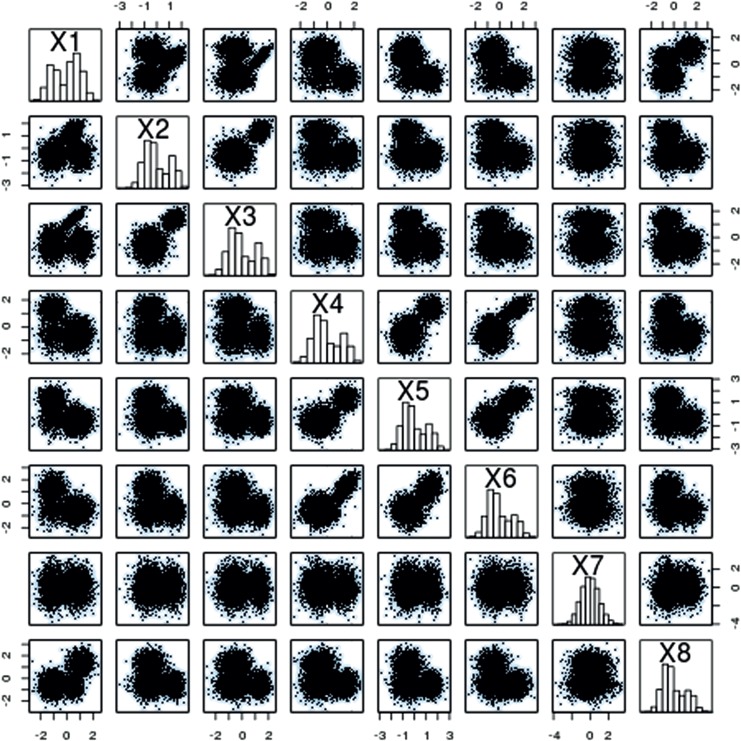
Pairwise scatter plots of a randomly selected subset of the $n=6000$ observations in the synthetic example.

\[\left ( \begin {matrix}1.5&0.6&0.9\\ 0.6&1&0.3\\ 0.9&0.3&0.8 \end {matrix}\right ) , \left ( \begin {matrix}1&0.7&0.9\\ 0.7&1.5&0.3\\ 0.9&0.3&2 \end {matrix}\right ) ,\]

respectively, the remaining dimensions have zero covariances with any other variables and variances 5. The covariance matrix for component 4 is $5I.$ Figure 1 displays standardized data.

The MCMC-based posterior from analysis with a mixture of at most 16 multivariate normals strongly identifies four effective components; discriminative summaries for the dominant first 3 appear in Table 1. For each of the first 3 components, these summaries identify a single but component-specific variable as being relevant and sufficient to achieve an overall average classification probability at 95% accuracy; they also indicate the extent to which—on this interpretable scale—additional variables can be expected to improve this, reaching $\sim$99% with two variable subsets that are again different across components. In contrast, RSM selects all the variables except $x_6,x_7$ (a union of the DIME selected variables), with no insights into relative discriminative information of variable subsets across components.

**Table 1.** Accuracy $A_c(h)$, $(c = 1:3),$ in the synthetic example, variables are ordered according to the forward search based on discriminative accuracy $A_c(\ast )$ or RSM

$Step$	1	2	3	4	5	6	7	8
Variable	$8^\ast$	1	$3^\dagger$	2	6	5	4	7
$A_1(h)$	0.956	0.986	0.999	1.000	1.000	1.000	1.000	1.000
$\tau _{1+ }(h)$	0.897	0.973	0.999	0.999	1.000	1.000	1.000	1.000
$\tau _{1-}(h)$	0.021	0.009	0.000	0.000	0.000	0.000	0.000	0.000
$\Delta _{1}(h)$	0.049	0.012	0.000	0.000	0.000	0.000	0.000	0.000
$\Delta _{-1}(h)$	0.050	0.022	0.001	0.000	0.000	0.000	0.000	0.000
Variable	$3^\ast$	$2^\dagger$	1	4	6	8	5	7
$A_2(h)$	0.985	0.999	1.000	1.000	1.000	1.000	1.000	1.000
$\tau _{2+ }(h)$	0.970	0.999	1.000	1.000	1.000	1.000	1.000	1.000
$\tau _{2-}(h)$	0.010	0.001	0.000	0.000	0.000	0.000	0.000	0.000
$\Delta _{2}(h)$	0.013	0.001	0.001	0.000	0.000	0.000	0.000	0.000
$\Delta _{-2}(h)$	0.022	0.001	0.001	0.001	0.000	0.000	0.000	0.000
Variable	$4^\ast$	$5^\dagger$	6	1	3	2	8	7
$A_3(h)$	0.980	0.999	0.999	1.000	1.000	1.000	1.000	1.000
$\tau _{3+ }(h)$	0.958	0.997	0.999	1.000	1.000	1.000	1.000	1.000
$\tau _{3-}(h)$	0.012	0.001	0.000	0.000	0.000	0.000	0.000	0.000
$\Delta _{3}(h)$	0.019	0.001	0.000	0.000	0.000	0.000	0.000	0.000
$\Delta _{-3}(h)$	0.027	0.002	0.001	0.001	0.000	0.000	0.000	0.000
Variable	1	2	4	8	5	3	6	7
$RSM$	0.312	0.580	0.618	0.646	0.669	0.681	0.682	0.681

*Note.* For the former, variables indicated by $^\dagger$ would terminate the forward search for discriminatory variables if we choose to do so based on a minimal change of 0.01 in accuracy. We now also indicate by $^\ast$ the last variable identified to define a minimal set with absolute accuracy (average classification probability) $A_c(h)\geqslant 0.95$, if this is achieved. We underline the index of the last variable entered using RSM.

## 5. Discriminatory markers in flow cytometry analysis

Multi-parameter flow cytometry can measure 15 or more variables—biological phenotypic or functional markers—on thousands of cells per second; it is a routine biological assay used in basic and clinical research laboratories worldwide. The primary use of flow cytometry data is in identifying subpopulations within large data sets that represent different regions of the multivariate marker space that relate to differentiation of cells and their biological function. Multivariate mixture models are increasingly used (Chan *and others*, 2008; Lin *and others*, 2013) and the interest in identifying relevant subsets of markers, addressing the general questions of discriminative subsets posed in Section 1, is fundamental to both analysis of experimental results and to the design and selection of marker variables for future studies.

**Fig. 2.**
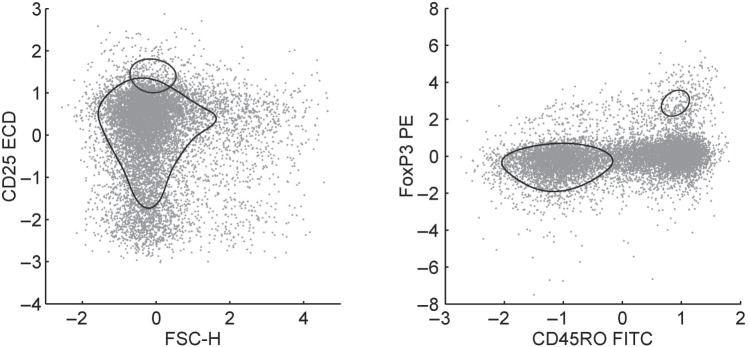
Scatter plots of standardized flow cytometry levels for 4 of the 12 marker variables, showing a randomly selected subsample of 10,000 cells. To highlight basic aspects of the mixture model analysis, the scatters are overlaid with contours of the corresponding two-dimensional margins of the two mixture components with highest estimated probabilities. Purely for display here, the specific estimate of the pdf is based on the mixture model with plug-in parameters defined by the posterior mode computed using the Bayesian EM algorithm, followed by component aggregation to identify the non-Gaussian subtypes displayed. The area of each contour displayed is approximately proportional to the corresponding posterior modal estimate of the resulting component probabilities.

We give an example in which a subset of variables is biologically known to define a scientifically interesting subpopulation. The study is an applied proof-of-concept, but also turns out to be more interesting biologically as we identify somewhat different marker subsets that have been commonly believed to be key. In this study, we are interested in regulatory T cells (Tregs), a specialized subtype of T cells that are critical to the maintenance of immune cell homeostasis and tolerance to self-antigens. We analyzed data from three replicate samples of peripheral blood mononuclear cells from the same donor to identify the minimal marker subset needed to discriminate Tregs from all other cellular subtypes. Each data set comprises $>1$ million observations coming from multiple cellular subtypes of which Treqs are just one subpopulation, and the $p=12$ scatter and fluorescent markers used in the analysis were FSC-A, FSC-H, SSC-A, vAmine (viability dye), CD3 V500, CD4 PerCP-Cy55, CD45R0 FITC, CD25 ECD, CD127 PE-Cy5, FoxP3 PE, Helios A647, and CD39 PE-Cy7. While FoxP3 is a master regulator in the development and function of Tregs and the most specific single Treg marker, it requires intra-nuclear staining to detect and the use of FoxP3 is not compatible with obtaining viable Tregs for functional studies using fluorescent activated cell sorting (FACS). Therefore, one major interest is to evaluate the extent to which FoxP3 is indispensable in identifying Tregs. Aspects of the data in four important marker variable dimensions, in displays that also illustrate—dimensional projections of some particular non-Gaussian cellular subtypes, appear in Figure 2.

**Fig. 3.**
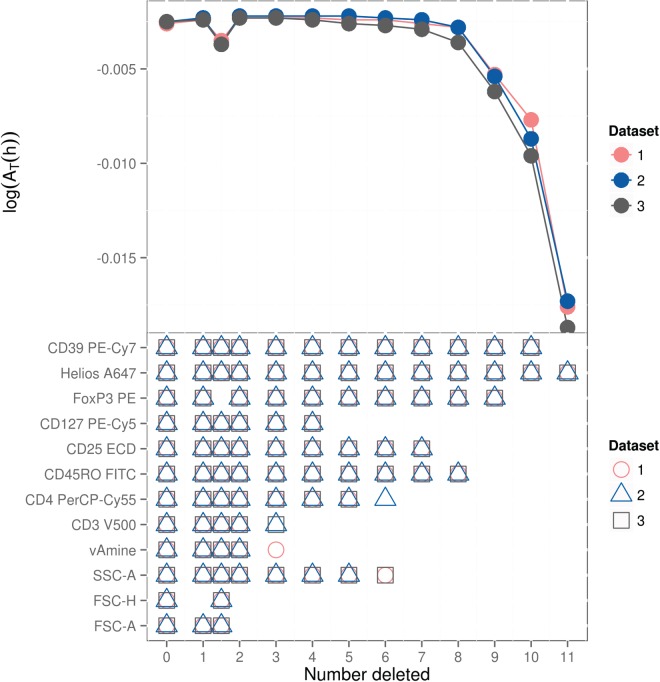
Summary discriminative measures for selected marker variable subsets in the analysis of Tregs flow cytometry data. The number of markers is decreased one at a time from left to right, with the best discriminative subsets indicated by different colors/symbols for each of the three data sets. The corresponding accuracy measures—plotted on the log scale—are shown at the top. The loss in accuracy when deleting FoxP3 is also shown—plotted, simply for convenience, at 1.5 on the horizontal axis.

Analysis used an upper bound of $J=160$ on the number of Gaussian components with the strategy of identifying subtypes by aggregating components around modes of attraction; see Supplementary material available at *Biostatistics* online. We remark that this aggregation strategy is now becoming standard in mixture model analysis in this application area. Repeat posterior mode search via the Bayesian EM algorithm of Supplementary material available at *Biostatistics* online identified a highest posterior mode used to initialize the MCMC, followed by component relabeling and aggregation to define possibly non-normal components ($C$ = 88, 86, and 123 for the three samples, respectively). From the posterior summaries resulting, we first identified a component corresponding to the Tregs cellular subtype based on biological knowledge; Tregs are defined as having high values in FoxP3, CD25, CD3 and CD4, and low values in vAmine and CD127. We use $c=T$ to denote the Treg subpopulation and evaluate markers for their ability to discriminate Treg cells from the remaining components– some of which are identifiable cellular subtypes– represented by the posterior. We computed MCMC-based posterior means of the probabilistic classification accuracy measures $A_T(h)$ for *all possible subsets* $h\subseteq {1:12}$; Figure 3 reports a summary selection, including the most discriminative subset of $k$ markers for each $k=1,\ldots ,12$.

Very good discrimination of Tregs is obtained using as few as four markers, with only modest increases in accuracy as markers are added; this is relevant for future studies to isolate only Treg cells with a reduced set of markers. The smallest subset of markers to identify Tregs in the absence of FoxP3 is CD39 and Helios with an accuracy $\sim$0.99. CD39 and Helios markers are known to characterize functionally active and thymic-derived Tregs, respectively (Borsellino *and others*, 2007; Thornton *and others*, 2010), and so constitute the dominant Treg population in these samples. However, as shown in Figure 3, dropping FOXP3 results in a small but significant decrease in accuracy, confirming that FOXP3 is an important marker characterizing Tregs.

## 6. Additional comments

The utility and effectiveness of the discriminative information and probabilistic classification measures introduced here are clear in both the synthetic and real data examples. Based on discrimination of mixture components via measures of concordance, the approach is intuitive and natural; the ties to misclassification rates provides additional theoretical insights and a map to the intuitive probability scale for evaluating and comparing variable subsets.

Coupled with existing approaches and software tools for posterior mode search and posterior simulation in multivariate mixtures, the approach extends the toolbox of statistical discrimination and classification for studies aiming to dissect the roles played by variables, both individually and in association with other variables, in the determination of discrimination of mixture subpopulations. In contrast to variable selection approaches (e.g., Raftery and Dean, 2006; Kim *and others*, 2006), this new method overlays an existing mixture model analysis to explore and quantify the roles of subsets of variables, and so can be applied easily and routinely across analysis. Our examples show how it relates to and improves upon existing approaches. Further in comparison, the current approach is computationally accessible and scalable. The dissection of roles of variables cuts deeper than other methods in evaluating local discriminative roles of variables; that is, assessing subsets of variables for their roles on each subpopulation, rather than aiming to select one set of variables for all components. This is key in applications such as the flow cytometry study illustrated here, where different, generally small subsets of variables can characterize subpopulations, with some variables being irrelevant for discrimination of many components but critically relevant for others.

## Supplementary material

Supplementary material is available online at http://biostatistics.oxfordjournals.org.

## Funding

This research was partially supported by grants from the U.S. National Institutes of Health (RC1-AI086032, P30-AI06451) and the National Science Foundation (DMS-1106516). Any opinions, findings, and conclusions or recommendations expressed in this work are those of the authors and do not necessarily reflect the views of the NIH or NSF.

## Supplementary Material

Supplementary Data

## Footnotes

1 It can be easily shown that the expected classification rates are, respectively, equal to the corresponding expected true- and false-positive classification rates under a *uniform prior on threshold $\tau$*, i.e.,

\[\alpha _{c+ }=\int _0^1 \textrm {Pr}(\alpha _c^* (x)>\tau |x\sim f_c)\,{\rm d}\tau \quad \textrm {and}\quad \alpha _{c-}=\int _0^1 \textrm {Pr}(\alpha _c^* (x)\geqslant \tau |x\sim f_{-c})\,{\rm d}\tau .\]

Hence, the true-positive/false-positive terminology—that is typically associated with a test based on thresholding posterior probabilities for given $x$—is seen to be apt.
