# Supplementary material for: Discriminative variable subsets in Bayesian classification with mixture models, with application in flow cytometry studies
Source: Biostatistics. 2015 Jun 3;17(1):40–53. doi: 10.1093/biostatistics/kxv021 (PMC4679067; doi:10.1093/biostatistics/kxv021)
Supplement: Supplementary Data [file supp_17_1_40__index.html]

Discriminative variable subsets in Bayesian classification with mixture models, with application in flow cytometry studies — Supplementary Data 

# Discriminative variable subsets in Bayesian classification with mixture models, with application in flow cytometry studies

## Supplementary Data

Supplementary Data

**Files in this Supplementary Material:**

- Supplementary Data
