# Supplementary material for: Discriminative variable subsets in Bayesian classification with mixture models, with application in flow cytometry studies
Source: Biostatistics. 2015 Jun 3;17(1):40–53. doi: 10.1093/biostatistics/kxv021 (PMC4679067; doi:10.1093/biostatistics/kxv021)
Supplement: Supplementary Data [file supp_kxv021_kxv021supp.pdf]

Discriminative Variable Subsets in Bayesian Classification with Mixture Models, with Application in Flow Cytometry Studies

LIN LIN[1], CLIBURN CHAN[2] and MIKE WEST[3]

[1]Vaccine and Infectious Disease Division, Fred Hutchinson Cancer Research Center, Seattle, WA 98109, USA
[2]Biostatistics & Bioinformatics, Duke University Medical Center,Durham, NC 27710-2721 & Department of Statistical Science, Duke University , Durham, NC 27708-0251, USA
[3]Department of Statistical Science, Duke University , Durham, NC 27708-0251, USA

1 Mixture of Gaussians under truncated Dirichlet process priors

We use the truncated Dirichlet process Gaussian mixture model Ishwaran and James (2001) in which $p-$vector observations x follow the model

$$g(x|\Theta) = \sum_{j=1}^{J} \pi_j N(\mu_j, \Sigma_j) \tag{1}$$

with prior hierarchically defined as follows:

$$\begin{aligned}
&\pi_1 = V_1, \qquad \pi_j = (1-V_1), ..., (1-V_{j-1})V_j, \quad 1 < j < J,\\
&V_j \mid \alpha \sim B(1, a), \quad j = 1, ..., J-1,\\
&a \sim G(e, f),\\
&\mu_j \mid \Sigma_j \sim N(m, t\Sigma_j),\\
&\Sigma_j \sim IW(k+2, kK)
\end{aligned}$$

for specified hyperparameters (e, f, m, t, k, K) and some fixed (large) upper bound J on the number of effective components. Based on observing the random sample $x_{1:n} = \{x_1, ..., x_n\}$ we simulate the full posterior for $\Theta = \{\mu_{1:J}, \Sigma_{1:J}, V_{1:J-1}\}$ and latent variables $(a, z_{1:n})$ where $z_{1:n} = \{z_1, ..., z_n\}$ is the set of latent configuration indicators, viz. $z_i = j$ if, and only if, x_i comes from normal component j. The standard blocked Gibbs sampler (Ishwaran and James, 2001; Ji et al., 2009) for this model is effective, widely used and implemented in efficient serial and parallel code (Suchard et al., 2010; Wang et al., 2010).

2 Component relabelling in MCMC analysis of mixtures

To resolve the well-known component label switching problem (e.g. West, 1997; Stephens, 2000; Yao and Lindsay, 2009) our Gibbs sampler imposes a per iterate relabelling based on the efficient and effective method of Cron and West (2011). The code used for the analysis incorporates this as a default (Wang et al., 2010), and the essential idea is summarized here.

At each Gibbs iterate let M be the $n \times J$ binary classification matrix with elements $M_{ij} = 1$ where $j = \text{argmax}_{r=1}^{J}\{\pi_r(x_i)\}$ based on current values of the component posterior classification probabilities $\pi_r(x) = \pi_r f_r(x_i)/g(x_i)$ under equation (1). Let M_0 denote a reference classification matrix obtained this way but using parameters Θ obtained as highest posterior modes following a Bayesian expectation-maximization based search. At the current simulation iterate, relabel components as follows.

1. Reorder components so that $\pi_1 > \pi_2 > \cdots > \pi_J$, reordering $\mu_{1:J}, \Sigma_{1:J}$ and the columns of M accordingly.

2. Beginning with column 1 of M_0, find column r_1 of M such that the two columns have the best match:

$$r_1 = \text{argmax}_{j=1}^{J} \sum_{1=1}^{n} M_0(i,1)M(i,j).$$

   Delete column 1 from M_0 and column r_1 from M. Repeat to assign a match of column 2 of the original M_0 with r_2 of the original M. Continue this to define the complete assignment of columns $[r_1, ..., r_J]$ and use this to reorder $\pi_{1:J}, \mu_{1:J}, \Sigma_{1:J}$ and reassign the z_i accordingly.

3 Bayesian EM algorithm in Dirichlet process mixtures

Numerical search to identify modes of the posterior $p(\Theta|x_{1:n})$ uses a new expectation-maximization procedure for truncated Dirichlet mixture models, as follows. This extends the standard method treating the latent variables $(a, z_{1:n})$ as missing data, iterating over $t = 0, 1, \ldots,$ based on starting parameter values $\Theta^{(0)}$, as follows. At iterate $t+1$:

E-step: Define $Q(\Theta|\Theta^{(t)}) = \text{E}[\ \log\{p(\Theta, z_{1:n}, a|x_{1:n})\}|\ \Theta^{(t)}, x_{1:n}\ ]$.

For given parameters Θ, denote the posterior classification probabilities by $\pi_{ij} = \pi_j(x_i) = \pi_j N(x_i|\mu_j, \Sigma_j)/g(x_i|\Theta)$ and define $\hat{a} = \text{E}[a|\Theta, x_{1:n}] = (J+e-1)/(f - \sum_{j=1}^{J-1}\log(1-V_j))$. Then $Q(\Theta|\Theta^{(t)})$ is given, up to a constant, by

$$Q(\Theta|\Theta^{(t)}) = c + \sum_{j=1}^{J}\Big[\sum_{i=1}^{n}\pi_{ij}^{(t)}\log\{\pi_j N(x_i|\mu_j,\Sigma_j) + \log[p(\mu_j|\Sigma_j)p(\Sigma_j)]\}\Big]$$
$$+ \sum_{j=1}^{J-1}(\hat{a}^{(t)} - 1)\log(1 - V_j).$$

M-step: Compute $\Theta^{(t+1)} = \text{argmax}_{\Theta}\, Q(\Theta|\Theta^{(t)})$. Letting $c_j^{(t)} = \sum_{i=1}^n \pi_{ij}^{(t)}$, this yields the following, with index j running from $j = 1, \ldots, J$ except as noted for the V_j terms:

$$V_j^{(t+1)} = \min\{1,\ c_j^{(t)}/[\hat{a}^{(t)} - 1 + \sum_{r=j}^{J} c_r^{(t)}]\ \};$$

$$\pi_1{}^{(t+1)} = V_1^{(t+1)}, \qquad \pi_j{}^{(t+1)} = (1 - V_1{}^{(t+1)}) \cdots (1 - V_{j-1}{}^{(t+1)}) V_j{}^{(t+1)}, \quad j = 2, ..., J;$$

$$\mu_j^{(t+1)} = (m + tc_j^{(t)}\bar{x}_j)/(1 + tc_j^{(t)}) \quad \text{where} \quad \bar{x}_j = \sum_{i=1}^{n} \pi_{ij}{}^{(t)} x_i / c_j^{(t)};$$

$$\Sigma_j^{(t+1)} = S_j^{(t)}/(c_j^{(t)} + k + 2p + 3) \quad \text{where}$$

$$S_j^{(t)} = kK + c_j^{(t)}(\bar{x}_j - m)(\bar{x}_j - m)'/(1 + tc_j^{(t)}) + \sum_{i=1}^{n} \pi_{ij}^{(t)}(x_i - \bar{x}_j)(x_i - \bar{x}_j)'.$$

A key practical point to note is that an identified posterior mode will typically identify fewer than the maximum specified number of components, so providing an automatic indicator of effective number of components from a mode search. This arises when the M-step optimization over the V_j yields $V_j = 1$ for $j \geq J'$, for some $J' < J$.

4 Non-Gaussian component mixtures via aggregating normals

Given a set of parameters, whether posterior mode estimates or a sample from the posterior, for the Gaussian mixture of equation (1), we follow previous work (Chan et al., 2008; Finak et al., 2009) in defining subpopulations by aggregating proximate normal components. That is, identify $C \leq J$ subpopulations with index sets I_c containing components indices j for each subtype $c = 1 : C$. Then $\alpha_c = \sum_{j \in I_c} \pi_j$ and

$$g(x) = \sum_{c=1}^{C} \alpha_c f_c(x) \quad \text{where} \quad f_c(x) = \sum_{j \in I_c} (\pi_j/\alpha_c) N(x_i|\mu_j, \Sigma_j),\ c = 1, \ldots, C.$$

Grouping components into clusters can be done by associating each of the normal components with the closest mode of $g(x)$. By running an efficient modal search beginning at each of the μ_j we can swiftly identify the set of modes in $g(x)$ together with the indicators of which mode each normal component is attracted too. The number of modes so identified is C, taken as the realized number of subpopulations in the mixture.

Efficient numerical optimization uses the mode trace function for Gaussian mixtures. Define precision matrices $\Omega_j = \Sigma_j^{-1}$. Mode search start with iteration index $i = 0$ and a point x^0 in data space and then, for $i = 1, 2, ...$ iteratively computes

$$x^{i+1} = A(x^i)^{-1} \sum_{j=1}^{J} \gamma_j(x^i) \Omega_j \mu_j$$

where $A(x) = \sum_{j=1}^{J} \gamma_j(x)\Omega_j$ and $\gamma_j(x) = \pi_j N(x|\mu_j, \Sigma_j)$. This is a convergent local mode search that is broadly useful to quickly identify modes, antimodes and ridge lines between them in the

contours of Gaussian mixtures, and typically takes just a few iterates. A second derivative of $g(x)$ evaluated at any identified stationary point then identifies it as a mode or antimode. Rather than being interested in all modes of $g(x)$, we are here only interested in those that define basins of attraction for the mixture components in order to find the sets I_c of component indicators related to different modes. Hence we run this numerical search J times, initializing at $x^0 = \mu_j$, $j = 1 : J$ in turn, and record the unique modes so identified as well as the sets I_c of Gaussian components attracted to each in this search.

5 Approximations to α_{c+} and α_{c-}

As a simple but illuminating example, take $p = 1 : 10$, $C = 2$, $\alpha_1 = \alpha_2 = 0.5$, $f_1(x) = N(x|0_p, I)$ and $f_2(x) = N(x|m_p, I)$ for some $m \neq 0$. In this special case, $\Delta_c = \Delta_{-c} = \exp(-p \times m^2/4)$ and $\tau_{c-} = 1 - \tau_{c+}$ where $\tau_{c+} = 1/(1 + \exp(-p \times m^2/4))$ for each $c = 1, 2$. Supplementary Figure 1 plots these values for $c = 1$. We also show Monte Carlo estimates of the expected posterior classification probabilities α_{c+}, α_{c-}, computed by importance sampling using 10,000 draws from a Cauchy importance sampling distribution.

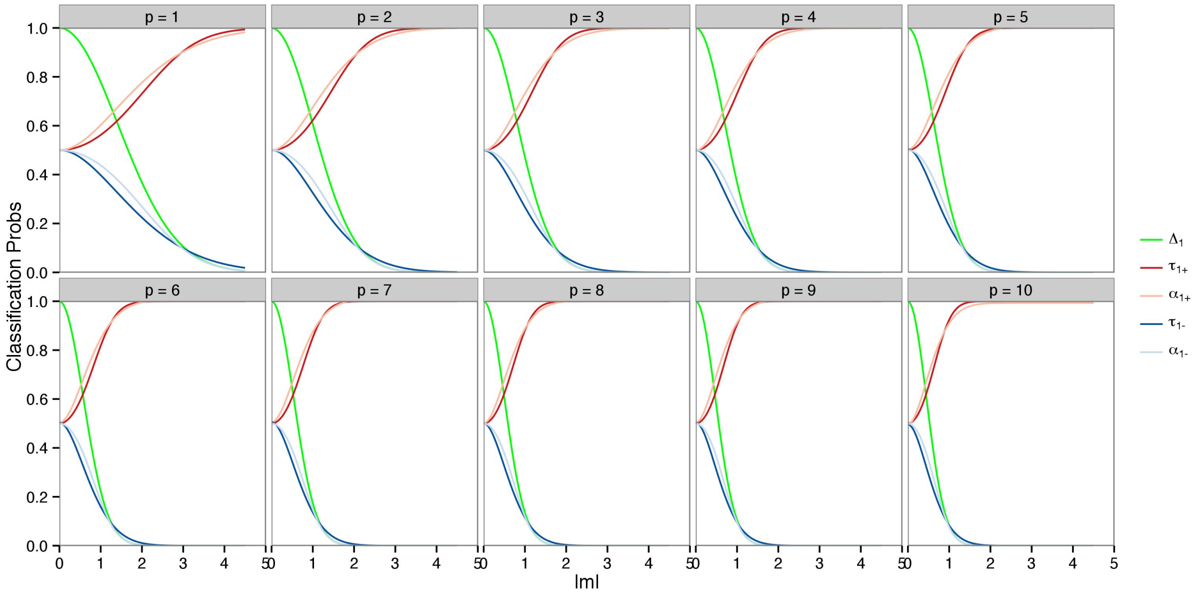

Supplementary Figure 1. Example with p ranging from 1 to 10, $C = 2, \alpha_c = 0.5$ for each $c = 1, 2$, and where $f_1(x) = N(x|0_p, I)$ and $f_2(x) = N(x|m_p, I)$. In this special case, $\Delta_c = \Delta_{-c} = \exp(-p \times m^2/4)$ and $\tau_{c-} = 1 - \tau_{c+}$ with $\tau_{c+} = 1/(1 + \exp(-p \times m^2/4))$ for each $c = 1, 2$.

6 Estimation of discriminative measures

If all mixture components of interest are normal, the effective number of components is C and each of the component densities $f_c(x)$ is normal. Then each DIME measure Δ_* is easily computable based on given mean vectors and variance matrices for components, and hence we can easily compute discriminative probabilities given the α_c parameters. Posterior analysis using MCMC methods

(see supplementary material and references) generates posterior samples of all model parameters, including C, and so we can directly compute the corresponding posterior samples for all discriminative measures of interest. Summary estimates of the Δ_*, τ_* and A_* quantities are based on approximate posterior means from these MCMC outputs in our examples below.

It is also of interest to consider plug-in estimates of the parameters based on posterior modes for the mixture model parameters. The supplementary material also details a new Bayesian expectation-maximization algorithm for finding posterior modes in truncated Dirichlet process mixtures of normals. This is an efficient numerical search strategy that can be run from multiple starting values to determine posterior modes. We use this to compare the resulting plug-in estimates of discriminative quantities with their MCMC-based posteriors and approximate posterior means; the Bayesian EM algorithm is also useful for quickly generating initial parameter values as starting values for the standard MCMC. Both MCMC and the Bayesian EM algorithm are particularly effective for problems with larger dimensions, numbers of components and sample sizes when exploiting the parallelization opportunities using GPU implementations (Suchard et al., 2010; Wang et al., 2010).

Under the contexts where practically relevant component densities f_c may have quite non-Gaussian forms. In the flow cytometry study, biologically relevant components are assumed to represent distributions of cell surface markers on specific cellular subtypes and these can exhibit markedly non-normal forms. Here we use the emerging standard strategy of assuming each $f_c(x)$ is itself a mixture of multivariate normals, i.e., $g(x)$ is a mixture of mixtures (Cao and West, 1996; Chan et al., 2008; Frelinger et al., 2010; Finak et al., 2009). This is operationally defined by setting $g(x)$ to be a mixture of multivariate normals with a large number of components, again utilizing the inherent parsimony of the Bayesian truncated Dirichlet process mixture to automatically cut-back to smaller numbers of components deemed relevant by the data. Then, given any set of components and their parameters, we use the modal aggregation strategy (e.g. Chan et al., 2008; Finak et al., 2009) to identify C sets of subsets of the normal densities and take each $f_c(x)$ as the implied conditional mixture of normals of one of these subsets; see Supplementary section 4. Whether using plug-in estimates from posterior modes or repeat evaluations using MCMC outputs, the Δ_* quantities are then easily evaluated since the underlying concordance measures $\delta_{a,b}$ between two mixtures of multivariate normals $f_a(x),\ f_b(x)$ with given parameters are analytically available.

7 Forward Search Algorithm

A simple forward search over subsets operates as follows. This applies separately– and in parallel– to each chosen component c of interest. Starting at $k = 0$ and with an initial empty variable subset $h \equiv h_{c0} = \emptyset$, move over a series of iterates, at each staging updating the variable subset. Suppose that at iterate $k \geq 0$ we have a current subset of variables $h_{ck} \subseteq \{1 : p\}$. Then at step $k + 1$:

1. For each $j \notin h_{ck}$, compute $A_c(j, h_{ck})$;
2. Identify $j^*_{ck} = \text{argmax}_{j \notin h_{ck}} A_c(j, h_{ck})$;
3. Update k to $k + 1$ and the current variable subset to (j^*_{ck}, h_{ck});
4. Continue to the next iterate, or stop.

We might simply continue this process until all variables are selected, or stop at point 4 if the increase in $A_c(*)$ is below a chosen threshold ϵ and/or if $A_c(*)$ exceeds a specified high probability. We can also address potential masking issues by modifications that have multiple branching subsets of selected variables by considering two or more different additional variables at step 3.

8 Two synthetic examples

8.1 A simple example

This simple example involves sample of size $n = 5,000$ drawn from $p = 3-$dimensional mixtures of $C = 2$ normal distributions. Only the first 2 variables carry primary discriminative information. The model and analysis allows up to 9 components using default, relatively vague priors in the truncated Dirichlet process mixture. The Bayesian expectation-maximization algorithm (Supplementary section 3) was run repeatedly from many random starting points. The posterior mode identified the correct number of components and parameters perfectly consistent with the known, true values underlying the synthetic data generation. MCMC analysis, as in Supplementary section 1 and 2, was initialized at the posterior mode identified by the Bayesian EM analysis, and run to generate posterior simulations of size 10,000 following additional burn-in iterates.

Supplementary Example 1. In this example, variables 1 and 2 together discriminate the 2 normal components while variable 3 is redundant. A data scatter plot appears in Supplementary Figure 2.

Supplementary Table 1 displays MCMC-based posterior means of discriminative measures. This clearly shows the $\tau_{c+}(h)$ and $\tau_{c,1}(h)$ correctly identify the first 2 variables as highly discriminative and that the 3rd variable is redundant. Note that τ_{c+} is close to 1 for $h = (1,2)$ and less than 0.9 for other subsets of just 1 or 2 variables; similarly, τ_{c-} is close to 0 for $h = (1,2)$ and greater than 0.1 for other subsets of just 1 or 2 variables; adding variable 3 to $(1,2)$ makes no practical change. In addition, the data was generated such that the difference between the two normal mean vectors (μ_1 and μ_2 for the two normal components) for variable 1 ($|\mu_1(1)-\mu_2(1)|$) is slightly larger than the difference for variable 2 ($|\mu_1(2) - \mu_2(2)|$), given that the variances are the same for both variable 1 and 2 among each of the normal component. Hence, Supplementary Table 1 also shows variable 1 alone discriminated better than variable 2 alone. Supplementary Figure 3 displays full posteriors for some of the $\tau_{c+}(h)$ and $\tau_{c-}(h)$ indicating very high concentration of posterior margins in this example.

In all cases, plug-in values based on the posterior modal parameters identified via the Bayesian EM search are very close to the MCMC-based posterior means for the DIME, $\tau_{c+}(h)$ and $\tau_{c-}(h)$ measures. In this example, all differences all well below 0.01 for the discriminative probabilities and most are much smaller. This is found in other examples, but as the mixture model dimension and complexity increase, we have found much more divergence between MCMC-based posterior means and plug-in values based on identified posterior modes. Hence as a routine we use MCMC, utilizing the EM search for initial exploratory analysis and initializing MCMC.

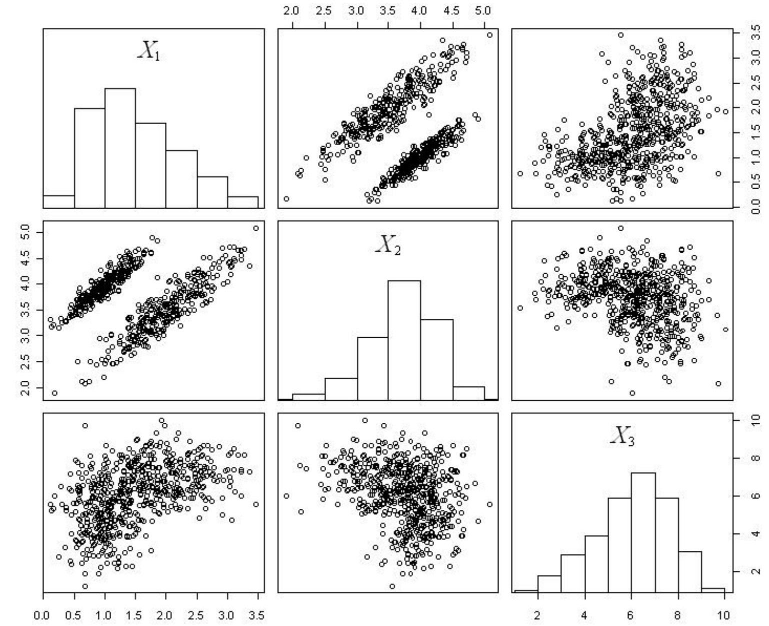

Supplementary Figure 2. Pairwise scatter plots of a randomly selected subset of the $n = 5,000$ observations in Supplementary Example 1. Dimensions 1 and 2 together discriminate the 2 normal components while dimension 3 is redundant.

Supplementary Table 1. MCMC-based posterior means for DIME measures and discriminative threshold probabilities $\tau_{c+}(h)$ and $\tau_{c-}(h)$ in Supplementary Example 1.

h	1,2,3	1,2	1,3	2,3	1	2	3
$\tau_{1+}(h)$	0.998	0.997	0.877	0.734	0.750	0.530	0.714
$\tau_{1-}(h)$	0.001	0.001	0.108	0.2380	0.173	0.356	0.371
$\Delta_1(h)$	0.002	0.003	0.145	0.373	0.345	0.911	0.413
$\Delta_{-1}(h)$	0.001	0.001	0.118	0.304	0.203	0.537	0.573
$\tau_{2+}(h)$	0.999	0.999	0.892	0.762	0.827	0.644	0.629
$\tau_{2-}(h)$	0.002	0.003	0.123	0.266	0.251	0.470	0.286
$\Delta_2(h)$	0.001	0.001	0.118	0.304	0.203	0.537	0.573
$\Delta_{-2}(h)$	0.002	0.003	0.145	0.373	0.345	0.911	0.416

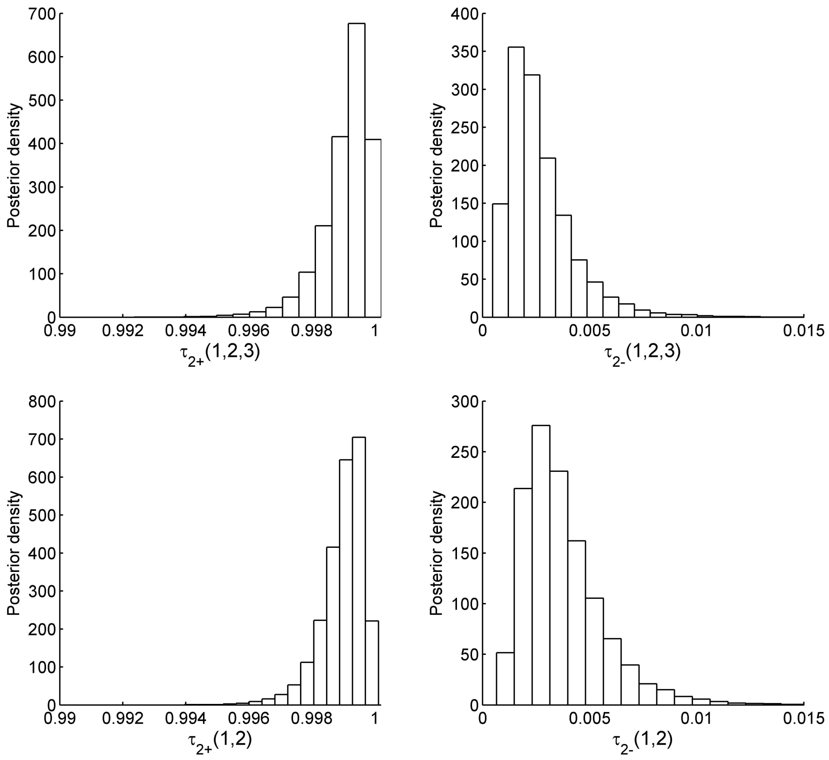

Supplementary Figure 3. MCMC-based histograms representing the posteriors for discriminative threshold probabilities in Supplementary Example 1. The upper two frames display posteriors for $\tau_{2+}(h)$ and $\tau_{2-}(h)$, respectively, when $h = (1, 2, 3)$, while the lower two frames show the corresponding posteriors when $h = (1, 2)$.

8.2 A more challenging mixture and comparison with related approaches

This more challenging mixture example comparing the analysis with the ridgeline-based separability measure (RSM) of Lee and Li (2012). Logistically, we follow the forward selected strategy and recommendations in Lee and Li (2012), evaluating variables to add to a current discriminatory subset if the increase in RSM exceeds 0.01 at each step, and stopping otherwise. MCMC analyses were initialized at the Bayesian EM-based posterior modes, and we generated posterior simulations of size 10,000 following additional burn-in iterates. For most direct comparison, RSM measures were evaluated using mixture model parameters estimated by MCMC-based posterior means.

Supplementary Example 2. This proof-of-principle example, where DIME and RSM approaches agree in identifying a single subset of discriminative variables for each of the components, follows Lee and Li (2012) in simulating 6,000 observations from the following $8-$dimensional distribution: the first two dimension are generated according to $1/3N((3, 9), I) + 1/3N((5, 6), I) + 1/3\text{Unif}([0, 8] \times [4, 12])$, where the uniform distribution serves to weaken the separation between the two primary normal components. The other six dimensions are non-informative, the variables being independent standard normals. Supplementary Figure 4 displays pairwise scatter plots of standardized data.

The model allowed up to 16 components and analysis used default, relatively vague priors. MCMC-based posterior outputs identify two main modes of concentration following aggregation of normal components (Supplementary section 4), with additional structure representing noise. More specifically, given the fitted 16-component mixture model, we identify modes by clustering the normal components into groups; this assigns each of the 16 components of the mixture to the closest mode of the fitted mixture model. Investigation of MCMC outputs (not shown) clearly show that the two components concentrate in regions consistent with those of the underlying distribution that generated the synthetic data. Discriminative analysis summaries are shown in Supplementary Table 2. Applying our stopping rule requiring a change of at least 0.01 on the classification probability scale for the accuracy measure $A_c(h)$ yields discriminative variable subsets $h = (1, 2)$ for each of the two components, correctly identifying the structure underlying the data. This agrees the RSM result that identifies $(1, 2)$ for both components simultaneously. This example highlights the ability of DIME-based analysis to perform as well as the existing method, while providing additional information: the table shows an average probability classification rate of about 92% for each component using variables $(1, 2)$, that variable 2 alone (or, in fact, variable 1 alone) would yield around 80-82% accuracies, and quite evidently the other variables only add noise to the discrimination.

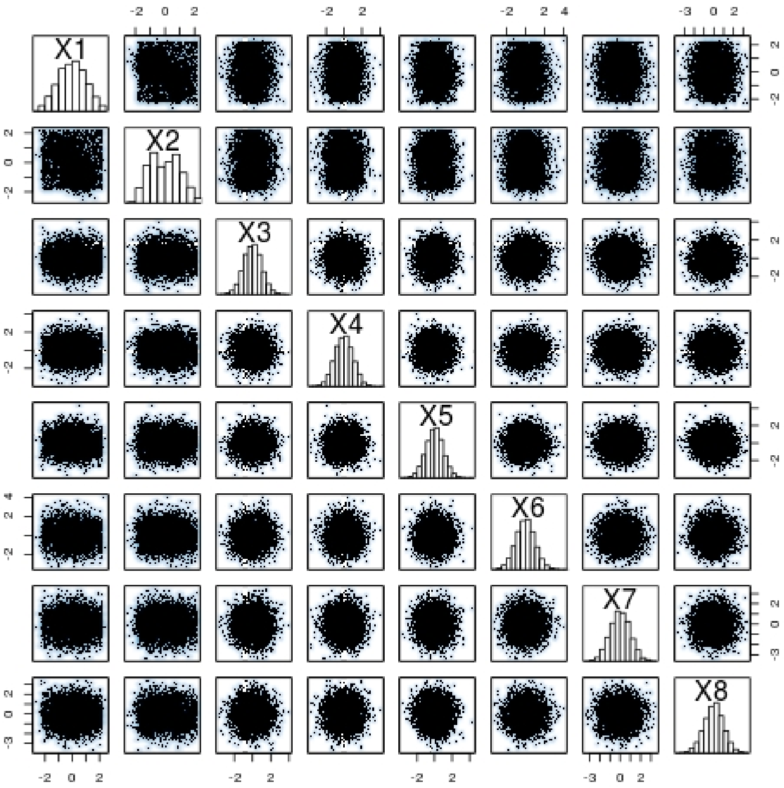

Supplementary Figure 4. Pairwise scatter plots of a randomly selected subset of the $n = 6,000$ observations in Supplementary Example 2.

Supplementary Table 2. Accuracy $A_c(h)$, $(c = 1, 2)$, in Supplementary Example 2. Variables are ordered according to the forward search based on accuracy $A_c(*)$ for DIME-based analysis to compare with the order and variables identified using the RSM approach. For each component, † indicates the last variable whose addition increases the accuracy probability by at least 0.01. RSM variables and values are defined as in Lee and Li (2012), computed at the posterior means of model parameters; underlining indicates the index of the last variable entered into the discriminative set using RSM.

Step:	1	2	3	4	5	6	7	8
Variable:	2	$1^{\dagger}$	6	4	7	3	8	5
$A_1(h)$:	0.808	0.928	0.928	0.928	0.927	0.927	0.926	0.924
$\tau_{1+}(h)$	0.792	0.923	0.923	0.923	0.922	0.921	0.920	0.918
$\tau_{1-}(h)$	0.179	0.069	0.068	0.068	0.068	0.068	0.069	0.070
$\Delta_1(h)$	0.232	0.073	0.074	0.074	0.075	0.076	0.077	0.079
$\Delta_{-1}(h)$	0.247	0.084	0.083	0.082	0.083	0.083	0.084	0.086
Variable:	2	$1^{\dagger}$	6	8	7	5	3	4
$A_2(h)$:	0.824	0.925	0.925	0.925	0.925	0.925	0.924	0.923
$\tau_{2+}(h)$	0.799	0.924	0.924	0.924	0.924	0.923	0.922	0.921
$\tau_{2-}(h)$	0.157	0.074	0.074	0.074	0.074	0.074	0.074	0.076
$\Delta_2(h)$	0.182	0.060	0.060	0.059	0.060	0.060	0.061	0.062
$\Delta_{-2}(h)$	0.258	0.111	0.111	0.111	0.111	0.111	0.112	0.114
Variable:	2	$\underline{1}$	5	8	3	7	6	4
RSM:	0.133	0.192	0.194	0.195	0.195	0.195	0.195	0.195

References

Cao, G. and West, M. (1996). Practical Bayesian inference using mixtures of mixtures. *Biometrics*, 52:1334–1341.

Chan, C., Feng, F., West, M., and Kepler, T. B. (2008). Statistical mixture modelling for cell subtype identification in flow cytometry. *Cytometry, A*, 73:693–701.

Cron, A. J. and West, M. (2011). Efficient classification-based relabeling in mixture models. *The American Statistician*, 65:16–20.

Finak, G., Bashashati, A., Brinkman, R., and Gottardo, R. (2009). Merging mixture components for cell population identification in flow cytometry. *Advances in Bioinformatics*, Article ID 247646.

Frelinger, J., Ottinger, J., Gouttefangeas, C., and Chan, C. (2010). Modeling flow cytometry data for cancer vaccine immune monitoring. *Cancer Immunology, Immunotherapy*, 59:1435–1441.

Ishwaran, H. and James, L. F. (2001). Gibbs sampling methods for stick-breaking priors. *Journal of the American Statistical Association*, 96:161–C173.

Ji, C., Merl, D., Kepler, T. B., and West, M. (2009). Spatial mixture modelling for unobserved point processes: Application to immunofluorescence histology. *Bayesian Analysis*, 4:297–316.

Lee, H. and Li, J. (2012). Variable selection for clustering by ridgeline-based separability. *Journal of Computational and Graphical Statistics*, 21:315–337.

Stephens, M. (2000). Dealing with label switching in mixture models. *Journal of the Royal Statistical Society, Series B*, 62:795–809.

Suchard, M. A., Wang, Q., Chan, C., Frelinger, J., Cron, A. J., and West, M. (2010). Understanding GPU programming for statistical computation: Studies in massively parallel massive mixtures. *Journal of Computational and Graphical Statistics*, 19:419–438.

Wang, Q., Cron, A. J., Chan, C., Frelinger, J., Suchard, M. A., and West, M. (2010). CPU and GPU code for Bayesian mixture modelling. http://www.stat.duke.edu/research/software/west/gpu/. Department of Statistical Science, Duke University.

West, M. (1997). Hierarchical mixture models in neurological transmission analysis. *Journal of the American Statistical Association*, 92:587–606.

Yao, W. and Lindsay, B. G. (2009). Bayesian mixture labeling by highest posterior density. *Journal of the American Statistical Society*, 104:758–767.
